# Radiotherapy regimens and concurrent Cabergoline use for non-functioning pituitary neuroendocrine tumors: a large, single-center cohort

**DOI:** 10.1007/s12020-025-04473-8

**Published:** 2026-02-03

**Authors:** Geovanne Pedro Mauro, Lucas Gonçalves Rebello, Leila Maria Da Róz, Vinicius de Carvalho Gico, Eduardo Weltman, Evandro César de Souza, Rafael Loch Batista, Malebranche Berardo Carneiro da Cunha Neto, Rosangela Correa Villar

**Affiliations:** 1 Department of Radiology and Oncology – Discipline of Radiotherapy- Faculdade de Medicina USP, Universidade de Sao Paulo, Sao Paulo, SP Brazil; 2 Department of Radiation Oncology, Instituto do Câncer do Estado de São Paulo (ICESP), Medical School of Sao Paulo University, Sao Paulo, Brazil; 3 Department of Neurology – Discipline of Neurosurgery- Faculdade de Medicina FMUSP, Universidade de Sao Paulo, Sao Paulo, SP Brazil; 4 Neuroendocrine Unit, Division of Endocrinology and Metabolism, Hospital das Clínicas, University of São Paulo Medical School, São Paulo, Brazil

**Keywords:** Pituitary adenomas, Radiosurgery, Radiotherapy, Stereotactic

## Abstract

**Background:**

The standard treatment for non-functioning pituitary neuroendocrine tumors (PitNET) consists in transsphenoidal surgical resection, however when progression or recurrence happens, additional treatments like stereotactic radiotherapy (SRT) and dopaminergic agonists can be used. In this article we aim to describe and quantify the impact of SRT regimen and dose, as well as the use of concurrent cabergoline, on local tumor control and toxicities.

**Methods:**

This is a retrospective cohort of patients with non-functioning PitNET treated between 2008 and 2023 with LINAC-based SRT on different dose regimens with or without cabergoline, in a single university hospital.

**Results:**

One-hundred twenty-six (126) patients were assessed. Median follow-up was 62.6 months (7.1 – 135.8 months). Most patients already had a visual deficit (65.1%) or hormonal dysfunction (52.4%) before SRT. Lesion size (median larger diameter is 2.5cm) and younger age were correlated with the use of fractionated SRT (FRT) (respectively, p>0.001 and p=0.006). 5 year control rate was 96.8%. For single-dose radiosurgery (SRS), they were 90.5% and 57.2 months, respectively. For FRT, 66.7% and 44.9 months, respectively. Cabergoline use did not influence results. There were no differences in new hormonal deficits among groups. Symptomatic radionecrosis was reported in only one patient. Strokes and CNS bleeding were reported in three patients. There were no secondary malignancies reported.

**Conclusion:**

No difference was found between SRS and FRT, the usage of cabergoline did not affect response. SRT brings good control with low toxicity in non-functional PitNET. However further prospective research is needed.

## Background

Non-functioning pituitary neuroendocrine tumors (PitNET) [1], formerly called non-functioning pituitary adenomas, are a large share of pituitary lesions, one that is often diagnosed latter than their secretive counterparts [2]. The standard treatment is focused on transsphenoidal surgical resection, but some patients may present residual tumors or progressive disease after one or more surgical approaches [3].

However, after disease progression there are no current standard recommendations for residual tumors. In such cases, radiotherapy and medical treatments can be considered based on patient profile [3]. The optimal radiotherapy regimen is not known, and multiple different techniques can be used with varying results [4]. Regarding medical treatments, dopaminergic agonists (mostly cabergoline) have been proposed to prevent tumor progression [5], also with mixed results [6]. Cabergoline has also been proposed as the primary treatment of NF-PitNETs, but the medical literature on this topic is still incipient [7, 8]. Since cabergoline seems to be a safe option in the NF-PitNET management, it has been tried in some cases to avoid SRT [9, 10]. The synergy between SRT and cabergoline, as well as timing for each treatment, has not been described previously to our knowledge.

In this report, our primary objectives are to comprehensively describe and quantify the impact of stereotactic radiotherapy (SRT) regimen and dose, and if the use of concurrent cabergoline would impact on progression-free survival and SRT-related toxicities.

## Patients and methods

All patients with a clinical and histopathological diagnosis of non-functioning PitNETs who were treated in the radiotherapy department of a large, academic hospital were assessed. All patients were treated between July 2008 and October 2023. Patients that were followed less than six months were excluded. All were treated in a LINAC-based setting.

Inclusion criteria were patients with no evidence of pituitary hormonal production and according to the immunohistochemistry (IHC) analysis.

SRT dose groups were divided between patients that were treated with single fraction and different doses of fractionated SRT. Group 1 was treated with single-dose stereotactic radiosurgery (SRS) with doses of 15 to 22 Gy; Group 2 received lower doses of RT (48.6 –50.4 Gy) in a hypofractionated regimen of at least 27 fractions of 180 cGy (FRT); and Group 3 received 54 Gy in a hypofractionated regimen of 30 fractions. The choice of method (SRS or FRT) was decided in a multidisciplinary tumor board consisting of radiation oncologists, radiologists, neurosurgeons, and medical physicists. The choice between methods was based on total volume and proximity to optic chiasm to respect dose constraints.

Concurrent cabergoline use was considered if patients had been using the medication for at least two weeks before the start of treatment and continued taking it throughout the SRT course. Its use, as altered hormone levels, were consistently assessed by the endocrinology team throughout follow-up.

Follow-up regarding tumor progression was done by MRI. This imaging study was requested twice a year for the first two years and annually afterwards. Survival was calculated from date of first RT treatment. Response was assessed by RECIST 1.1 guidelines.

Visual, nervous, and hormonal assessment were done every six months during follow-up. Campimetry was favored for all patients, but it was not mandatory. Endocrine function was assessed pre- and post-SRT using serum levels of free thyroxine (fT4), thyroid-stimulating hormone (TSH), cortisol, adrenocorticotropic hormone (ACTH), prolactin (PRL), growth hormone (GH), insulin-like growth factor-1 (IGF-1), follicle-stimulating hormone (FSH), luteinizing hormone (LH), testosterone, and estradiol. Hypopituitarism was characterized following criteria previously published [11].

Statistical analyses were conducted to evaluate survival outcomes and group differences. The Fisher’s exact test and chi-square test were applied to compare categorical variables across groups. Survival time was defined as the interval from the date of radiotherapy to the last follow-up or event and was assessed using the Kaplan-Meier method. The primary endpoints included overall survival (OS) and progression-free survival (PFS). Toxicity outcomes included survival free of new visual deficits (NVDFS), survival free of new hormonal deficits (NHDFS), and the incidence of radionecrosis, stroke, CNS bleeding, and second malignancies. Univariate comparisons of survival curves were performed using the log-rank test. Local ethics approved this retrospective study. This research was not founded by any funding agencies in the public, commercial, or not-for-profit sectors.

## Results

One-hundred twenty-six (126) patients were included. Mean age at treatment was 51.7 years. Patients were well balanced between genders (male/ female: 49.2/ 50.8%). Most patients already complained of visual deficit by first visit to RT clinic (65.1%) and met criteria for hypopituitarism (52.4%). Only 45 (35.7%) had campimetry assessment before RT, of those only 9 (20.0%) did not report any visual fields deficits.

Demographic characteristics were assessed. Results for those can be seen in Table 1.

**Table 1:** Demographic features

Patients’ characteristics		*N* = 126	
Age			
< 50 years	52 (41.3%)		
> 50 years	74 (58.7%)		
Gender			
Male	62 (49.2%)		
Female	64 (50.8%)		
Size			
< 3 cm	80 (63.5%)		
> 3 cm	46 (36.5%)		
Type			
Null cell	105 (83.3%)		
Silent gonadotrophic	21 (16.7%)		
Previous visual deficit			
No	44 (34.9%)		
Yes	82 (65.1%)		
Altered campimetry			
No	36 (28.6%)		
Yes	9 (7.1%)		
No previous campimetry	81 (64.3%)		
Compressed chiasm (MRI)			
No	50 (39.7%)		
Yes	76 (60.3%)		
Previous hypopituitarism			
No	60 (47.6%)		
Yes	66 (52.4%)		

Median lesion size was 2.8 cm (range 0.7 to 7.8 cm; interquartile interval 25% 1.8 cm to 75% 3.7 cm). All patients had undergone previous pituitary surgery. Median interval between first surgery and RT treatment was 27.7 month (3.0–231.9 months). Mean number of previous surgeries was 1.75 surgeries (1–5 surgeries) before referral to RT. Most patients were diagnosed with compressed or dislocated optic chiasm (76/ 60.3%) before RT. There were no re-irradiation patients.

We assessed the difference between RT groups. Group 1 consisted of nine (*N* = 9) patients with median dose of 18 Gy (15–22 Gy). Groups 2 (*N* = 102) and 3 (*N* = 15) were treated with a median dose of 50.7 Gy (45–54 Gy). Dose to optic chiasm were constrained to 54 Gy, nevertheless three patients received doses superior to 54 Gy to the structure. Mean maximum RT dose to chiasm was 48.5 Gy (range 41.8 to 57.4 Gy; interquartile interval 25% 50.1 Gy – 75% 52.6 Gy). Among RT groups, the biggest differences were that patients who were treated with single fraction SRS had lower rates of previous visual deficit (*p* = 0.001), lower rates of altered campimetry before treatment (*p* = 0.009), and lower rates of chiasmatic compression (*p* = 0.006). Different RT treatments did not impact outcomes.

We also assessed differences based on the concomitant use of cabergoline. Median dose of cabergoline prescribe was 0.55 mg/ week. Most patients (85/ 67.4%) did not receive dopaminergic agonist treatment during follow-up. However, approximately one-third of the participants used cabergoline concomitantly. No significant differences were observed between the cabergoline and non-cabergoline groups. Moreover, neither prior nor concurrent cabergoline use had an impact on clinical outcomes. The data regarding the comparison among groups are reported in Table 2.

**Table 2:** Baseline characteristics of patients stratified by concurrent Cabergoline use and radiotherapy regimens

*N* = 115	Concurrent cabergoline			Radiotherapy			
	No *N* = 107 (84.9%)	Yes *N* = 19 (15.1%)	*p*	Group 1 *N* = 9 (7.1%)	Group 2 *N* = 102 (81.0%)	Group 3 *N* = 15 (11.9%)	*p*
Age							
< 50 years	45 (42.1%)	7 (36.8%)	0.67	4 (44.4%)	42 (41.2%)	6 (40.0%)	0.97
> 50 years	62 (57.9%)	12 (63.2%)		5 (55.6%)	60 (58.8%)	9 (60.0%)	
Gender							
Male	56 (52.3%)	13 (68.4%)	0.09	4 (44.4%)	50 (49.0%)	10 (66.7%)	0.41
Female	51 (47.7%)	6 (31.6%)		5 (55.6%)	52 (51.0%)	5 (33.3%)	
Size							
< 3 cm	66 (61.7%)	14 (73.7%)	0.31	8 (89.9%)	63 (61.8%)	9 (60.0%)	0.29
> 3 cm	41 (38.3%)	5 (26.3%)		1 (11.1%)	39 (38.2%)	6 (40.0%)	
Type							
Null cell	90 (84.1%)	15 (79.0%)	0.52	8 (88.9%)	84 (82.4%)	13 (86.7%)	1.00
Silent gonadotrophic	17 (15.9%)	4 (21.0%)		1 (11.1%)	8 (17.6%)	2 (13.3%)	
Previous visual deficit							
No	39 (36.4%)	5 (26.3%)	0.39	8 (88.9%)	33 (32.3%)	3 (20.0%)	**0.001**
Yes	68 (63.6%)	14 (73.7%)		1 (11.1%)	69 (67.7%)	12 (80.0%)	
Altered campimetry							
No	8 (21.6%)	1 (12.5%)	0.48	3 (100%)	5 (13.5%)	1 (20.0%)	**0.009**
Yes	29 (78.4%)	7 (87.5%)		0	32 (86.5%)	4 (80.0%)	
No previous campimetry	70	11		6	65	9	
Compressed chiasm (MRI)							
No	43 (40.2%)	7 (36.8%)	0.78	8 (88.9%)	36 (35.3%)	6 (40.0%)	**0.006**
Yes	64 (59.8%)	12 (63.2%)		1 (11.1%)	66 (64.7%)	9 (60.0%)	
Previous hypopituitarism							
No	54 (50.5%)	6 (31.5%)	0.12	6 (66.7%)	50 (49.0%)	4 (26.7%)	0.14
Yes	53 (49.5%)	13 (68.5%)		3 (33.3%)	52 (51.0%)	11 (73.3%)	
Concurrent cabergoline							
No	-	-	-	9 (100%)	86 (84.3%)	12 (80.0%)	0.44
Yes				0	16 (15.7%)	3 (20.0%)	
RT groups							
I	9 (8.4%)	0	0.44	-	-	-	-
II	86 (80.4%)	16 (84.2%)					
III	12 (11.2%)	3 (15.8%)					

**Fig. 1:**
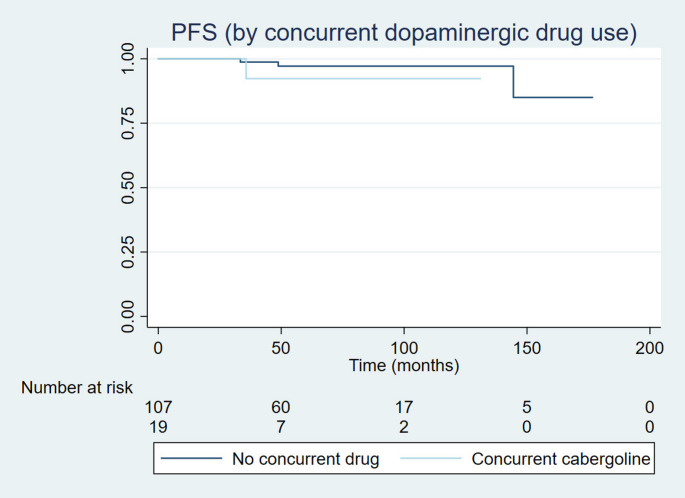
Progression-free survival according to dopaminergic medication (cabergoline) use. Median PFS for both groups were not reached

**Fig. 2:**
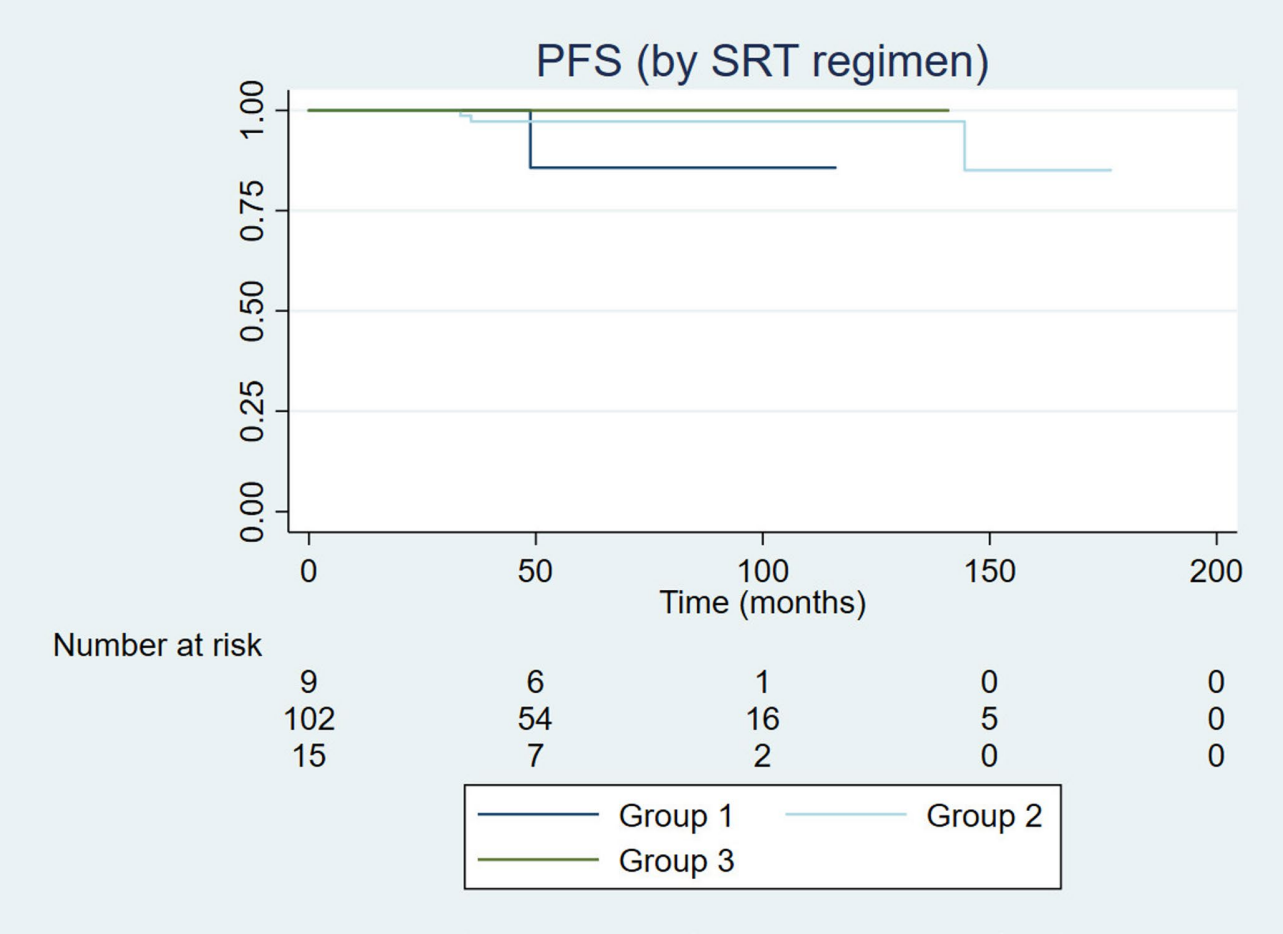
Progression-free survival according to RT technique and dose. Median PFS for all groups were not reached. Groups 1 was treated with SRS, Group 2 with low dose (< 54 Gy FRT, and Group 3 FRT with doses ≥ 54 Gy

Median follow-up was 62.4 months (6.6–176.6 months). There were two reported deaths. Median OS, PFS, and NVDFS were not reached. Median NHDFS was 89.6 months. Rates at 5 years for OS, PFS, NVDFS and NHDFS were 98.4%, 96.8%, 94.4%, and 80.9%, respectively. There was no difference in OS ([HR], 0.89; 95% CI, 0.33–23.5; *p* = 0.94), PFS ([HR], 0.20; 95% CI, 0.22–1.95; *p* = 0.17) (Figs. 1 and 2), NVDFS ([HR], 0.89; 95% CI, 0.17–4.45; *p* = 0.88), and NHDFS ([HR], 0.69; 95% CI, 0.33–1.46; *p* = 0.33) between SRT regimen groups. There was neither for the use of concurrent cabergoline, as in OS ([HR], 0.45; 95% CI, 0.0–77.5; *p* = 0.50), PFS ([HR], 3.34; 95% CI, 0.30–37.3; *p* = 0.32) (Figs. 1 and 2), NVDFS ([HR], 12.0; 95% CI, 0.0–81.1; *p* = 0.12), and NHDFS ([HR], 0.80; 95% CI, 0.28–2.27; *p* = 0.67). Regarding new hormonal deficits, 38 patients developed new pituitary hormonal deficit. Five developed only glucocorticoid deficiency, five only thyrotropic deficiency, ten only growth hormone deficiency. A total of 18 developed multiple pituitary hormonal deficiencies. Two patients were submitted to treatment with temozolomide, one before and one after SRT, both in Group 2, without the use of concurrent cabergoline.

Symptomatic radionecrosis was reported in only one patient, six years after SRT (dose to PTV was 50.4 Gy and to the chiasm was 49.9 Gy). Strokes and CNS bleeding were reported in three patients. The latency period between treatment and CNS bleeding/ strokes for those patients were 40.0, 49.4, and 105.3 months. There were no secondary malignancies reported.

Disease control was reported in 96.8% of patients by RECIST 1.1 criteria. All progression happened before 60 months of follow-up. There were one (0.7%) reported complete responses, partial responses were 30 (23.8%), stable disease were 91 (72.2%) and disease progression occurred in 4 (3.2%). No variable correlated to local control rate.

No variable impact neither OS nor NHDFS in the univariate analysis. The only variable to impact PFS was prior visual deficit (*p* = 0.02). Regarding NVDFS, previous symptomatic vision deficits correlated to new deficits after treatment, even asymptomatic ones (*p* = 0.01). No multivariate analysis was conducted based on these results. Results for univariate analysis can be seen in Table 3.

**Table 3:** Univariate analysis; performed using the log-rank test. No survival median values were reached

	*N*	OS		PFS		NVDFS		NHDFS	
		5 year rate (%)	*p*	5 year rate (%)	*p*	5 year rate (%)	*p*	5 year rate (%)	*p*
Concurrent cabergoline									
No	107	99.1	0.62	98.1	0.29	94.4	0.25	80.4	0.67
Yes	19	100		94.7		100		94.7	
RT groups									
I	9	100	0.80	88.9	0.32	100	0.88	77.7	0.62
II	102	99.1		98.0		95.1		80.4	
III	15	100		100		93.3		93.3	
Age									
< 50 years	52	100	0.22	98.1	0.77	100	0.08	84.6	0.78
> 50 years	74	98.6		97.3		91.9		79.7	
Gender									
Male	62	100	0.89	96.7	0.27	95.2	0.82	79.0	0.32
Female	64	98.4		98.4		95.3		84.3	
Size									
< 3 cm	80	100	0.73	97.5	0.48	96.3	0.48	86.3	0.94
> 3 cm	46	97.8		97.8		93.5		73.9	
Type									
Null cell	105	99.0	0.08	98.1	0.76	95.2	0.94	82.9	0.65
Silent gonadotrophic	21	100		95.2		95.2		76.2	
Previous visual deficit									
No	44	100	0.31	93.1	**0.02**	97.7	0.67	75.0	0.08
Yes	82	98.8		100		93.9		85.4	
Altered campimetry									
No	36	94.4	-	100	1.00	97.2	**0.01**	83.3	0.22
Yes	9	100		100		88.8		77.8	
No previous campimetry	81	98.7		96.3		95.1		81.5	
Compressed chiasm (MRI)									
No	50	100	0.30	96.0	0.24	98.0	0.55	80.0	0.40
Yes	76	98.7		98.7		93.4		83.0	
Previous panhypopituitarism									
No	60	98.3	0.91	96.7	0.41	98.3	0.12	75.0	0.06
Yes	66	100		98.5		92.4		89.4	

## Discussion

To our knowledge, this is the largest, retrospective cohort of exclusively non-functioning PitNET to be treated exclusively in a LINAC-setting to date. Retrospective analyses are always at risk of selection and register biases, but the size of this report makes it robust, and its data may be generalized. Amongst the novelties of our report are the exclusive use of LINAC-based and the comprehensive description of the prescription of cabergoline to patients undergoing SRT and the assessment of its timing. Although it is a large sample, most patients were treated with FRT. There was a limited number of patients that were submitted to single-fraction SRS and that may increase the risk of bias when comparing smaller groups.

Surgery is the cornerstone of the treatment of PitNETs. Most large publications on non-functioning PitNET describe surgical patients. The use of SRT after surgery is often reserved for progressive disease [12], as most patients may not require further treatment after first surgery [13]. SRT may improve local control, but there is no reported benefit of immediate adjuvant SRT over active surveillance after surgery, which could raise the number of patients submitted to SRT [14]. The necessity for SRT is very low, and most reports are under 20% [15–19], but can be higher in larger lesions or aggressive PitNETs [20]. Nevertheless, it which may not translate into more toxicities after treatment [21]. It is important to highlight that all patients reported here had their surgical options exhausted before SRT.

Regarding SRT techniques, most reports on PitNETs come from the experience with Gamma-Knife radiosurgery (GK). Historical and paramount publications on the matter are with the exclusive use of GK, reporting disease control of 87.3% [22] to 100% [23] with large follow-up [24–29]. Toxicities are also reported, most commonly new hormonal deficits of around 18–32% [30, 31]. Visual and neurological complications are less frequent, varying around 0% [32] to 13% [33]. The direct comparison between techniques cannot be compared outside a clinical trial. Our results of local control of 96.8%, new hormonal deficits of 29.4% and new visual deficits of 6.3% are compatible with previous GK publications. There are differences between both techniques, mostly regarding hot spots and planning techniques. Nevertheless, the results can be considered at least comparable.

One of our primary objectives was to determine the best SRT regimen in a LINAC-based setting. We compared the results of singe-dose SRS, and FRT with lower dose (40–50.4 Gy) or higher dose (54–56 Gy). We could not demonstrate superiority of any of those regimens in a LINAC-based setting for non-functioning PitNETs. Previous publications of smaller cohorts exclusively of non-functioning PitNETs and cohorts of patients with non-functioning and secretive lesions have shown similar results for LINAC-based SRT than those for GK, in terms of local control [34, 35] and toxicities [36, 37]. Previous publications also proposed that higher doses of FRT were unnecessary to achieve local control [38]. Another issue previously investigated is the comparison between singe-dose SRS and FRT, with mixed results [39–41]. Our results show similar conclusions that higher dose is not associated with better local control. It also shows that SRS is not superior to FRT, and the regimen should be chosen and tailored to the patients’ necessities and expectations.

Regarding the dose for FRT, it is important to highlight that our report and previously published data [38] have showed comparable results for doses between 45 and 50 Gy and the results for doses of 54 Gy and higher. We would suggest, as it is practice [2, 4] in most current guidelines, there is a lack of evidence favoring dose escalation in this disease and our results add to this conclusions and current practice.

Safety is a cornerstone for SRT regimen choice. The incidence of hormonal deficiencies after SRT has been previously demonstrated in larger retrospective cohorts of GK patients and must be kept in mind whenever prescribing SRT [42]. A noteworthy aspect of our report is that our cohort consisted exclusively of LINAC-treated patients with non-functioning PitNETs, where we applied a broader definition of pituitary hormonal deficiencies. In our study, the diagnosis was based on hormonal assessments rather than solely on the prescription of hormonal replacement therapy, as previously reported by Cordeiro et al. [34]. Neurological toxicities, however, are less dependent on SRT regimen [43–45] and may not impact quality of life [46].

The other primary endpoint of this report is to assess the impact of the concurrent use of dopaminergic agonists with SRT. Previous publications have reported some benefit [6, 11, 47] of cabergoline in non-functioning PitNETs management, and this may be an alternative to avoid or postpone SRT and its toxicities. They are however new and the drug have not been consistently prescribed for these patients in the past. The novelty in our report is that we did not find any beneficial association of cabergoline prior or concurrent to SRT. Previous smaller publications have suggested otherwise [48]. In this publication, the negative impact of combined treatment may come from the effect of cabergoline on cell cycle, which promotes an arrest at the radioresistant G1 phase. Nevertheless, that publication is small and comprised of both functioning and non-functioning PitNET. Another information that can be viewed as bias is the cut for concurrent use of cabergoline. This two-week period was used for three main reasons. First, it was the minimum interval for at least one half-life of the drug to be excreted. Second, the main reference we have on the manner is the very limited report of Takkar et al. (2020) [48]. Even though they haven’t reported what they would consider to be simultaneous delivery of cabergoline and SRT, they have stated that the smallest interval was 17 days. Third, because it usually takes two weeks between the first consultation for the radiation oncology department and treatment start, which could be consistently more accurate. We also haven’t demonstrated any enhancement in toxicities with the association of SRT and cabergoline. We discuss that cabergoline should be prescribed as necessary by the referring endocrinologist, without the concern for worse toxicities.

Despite providing novel data on the use of radiotherapy in the management of non-functioning PitNETs and being the first to assess the combined use of cabergoline in terms of radiotherapy efficacy and safety, this study has several limitations. It is a retrospective study, and the prescription of cabergoline was made by the attending physicians before the indication for radiotherapy, meaning that compliance with cabergoline use was not monitored. All included cases were unequivocally classified as non-functioning; however, the histological classification of these tumors did not follow the 2022 WHO criteria, limiting the evaluation of potential differences across tumor subtypes. Nonetheless, the low incidence of toxicities and the lack of impact of cabergoline use in most cases suggest that there are no subtle differences between non-functioning tumor subgroups regarding treatment outcomes.

## Conclusion

This is a large, retrospective cohort of non-functioning PitNET patients treated with SRT in a LINAC-setting. We could not demonstrate any benefit comparing single-dose SRS and FRT in this population. Higher doses of FRT also did not translate into local control and dose escalation should not be favored. The use of cabergoline, prior or during SRT, did not impact outcomes. Prospective data is needed.

## Data Availability

No datasets were generated or analysed during the current study.
